# Using administrative data to evaluate national policy impacts on child and maternal health: a research framework from the Maternal and Child Health Network (MatCHNet)

**DOI:** 10.1136/jech-2023-220621

**Published:** 2023-07-18

**Authors:** Emma Stewart, Sinead Brophy, Richard Cookson, Ruth Gilbert, Joanne Given, Pia Hardelid, Katie Harron, Alastair H Leyland, Anna Pearce, Rachael Wood, Ruth Dundas

**Affiliations:** University of Glasgow; Swansea University; University of York; University College London; Ulster University; University College London; University College London; University of Glasgow; University of Glasgow; Public Health Scotland; University of Glasgow

## Abstract

Reducing health inequalities by addressing the social circumstances in which children are conceived and raised is a societal priority. Early interventions are key to improving outcomes in childhood and long-term into adulthood. Across the UK nations, there is strong political commitment to invest in the early years. National policy interventions aim to tackle health inequalities and deliver health equity for all children. Evidence to determine the effectiveness of socio-structural policies upon child health outcomes is especially pressing given the current social and economic challenges facing policy makers and families with children. As an alternative to clinical trials or evaluating local interventions, we propose a research framework that supports evaluating the impact of whole country policies on child health outcomes. Three key research challenges must be addressed to enable such evaluations and improve policy for child health: i) policy prioritisation, ii) identification of comparable data, and iii) application of robust methods.

## Introduction

Giving every child the best start in life was identified as the highest priority recommendation of The Marmot Review, *Fair Society*, *Healthy Lives* [1]. Action to reduce health inequalities must begin before birth and continue through early childhood. UK Government strategies and policies identify childhood and pregnancy as crucial stages for policy intervention[2, 3]. Commitment to children’s rights and wellbeing is also evident across the different children’s strategies and policies of the 4 UK nations[4]. There is strong political will to invest in the early years yet surprisingly little evidence to substantiate the impact of national policies upon child and maternal health outcomes, or to understand the impact of funding cuts in key policy areas[5, 6].

In general, policy intervention in the early years makes sense economically; ‘Heckman’s curve’ graphically demonstrates how early investments in children result in the highest rate of fiscal return[7]. Nevertheless, robust outcome evaluations are essential to ensure investment is allocated to national policies that will improve child health and reduce health inequalities[8]. Evidence on impactful national policies is necessary to stop potentially harmful policies and promote those beneficial for health equity. Equally, there is a moral and ethical obligation[9] to tackle child health inequities, recognising the social justice aspects of child health and well-being across the life course. The evaluation of population level policies must therefore extend beyond health policies to include a wider range of upstream social, economic, and welfare policies that potentially affect child health[8, 10].

## Current Situation

In public health research, stronger evidence exists for individual-level clinical interventions compared to population-level social interventions with larger population health impacts[11]. This ‘inverse evidence law’, whereby the availability of good evidence tends to vary inversely with the requirement for it in the population served, also applies to national early childhood policies. To begin tackling this, there is a need for high quality evidence for universal or national policies that affect maternal and child health, and in turn impact on non-communicable diseases throughout the life course. Furthermore, even where strong evidence may exist for the overall benefits of some early years’ interventions, there is often a paucity of evidence around their impacts on health inequalities[10, 12]. Key to this is understanding the impacts of different models of eligibility, intensity, and uptake.

There are significant challenges to conducting national policy evaluations. Randomised controlled trials are rarely feasible or appropriate in this context as new policies are usually implemented across entire populations meaning it is difficult to identify suitable control groups. Instead, researchers must rely upon observational data that follow mothers and children over time, whether through recruited cohorts and/or administrative data, to compare the health outcomes of children or mothers that have naturally been exposed to different policies or contexts[13, 14]. Previous evaluations have included comparing children living in countries/regions with different policies, or examining how child and maternal health outcomes alter over time following policy change[15, 16].

A further challenge is accounting for other policy variations that exist between populations and that might change outcomes[17]. New methods and better data can help with this. Improvements in data quality, longevity, and depth, and increased capacity for whole country analysis of administrative data, offer a new and timely opportunity for national preventive policy evaluation. These data resources bring their own challenges[18–20]. Nevertheless, a major advance is the continuity of longitudinal data collection over time, which can be used to measure indicators in a dynamic policy environment. Natural experiments (NE) that focus on population-wide and system-wide approaches are a valuable piece of the public health toolkit[21, 22]. NE studies use a naturally occurring variation in policy exposure to identify the impact on an outcome of interest.

## Developing a Research Framework to Evaluate National Policies in Child and Maternal Health Using Administrative Data

**Figure 1:**
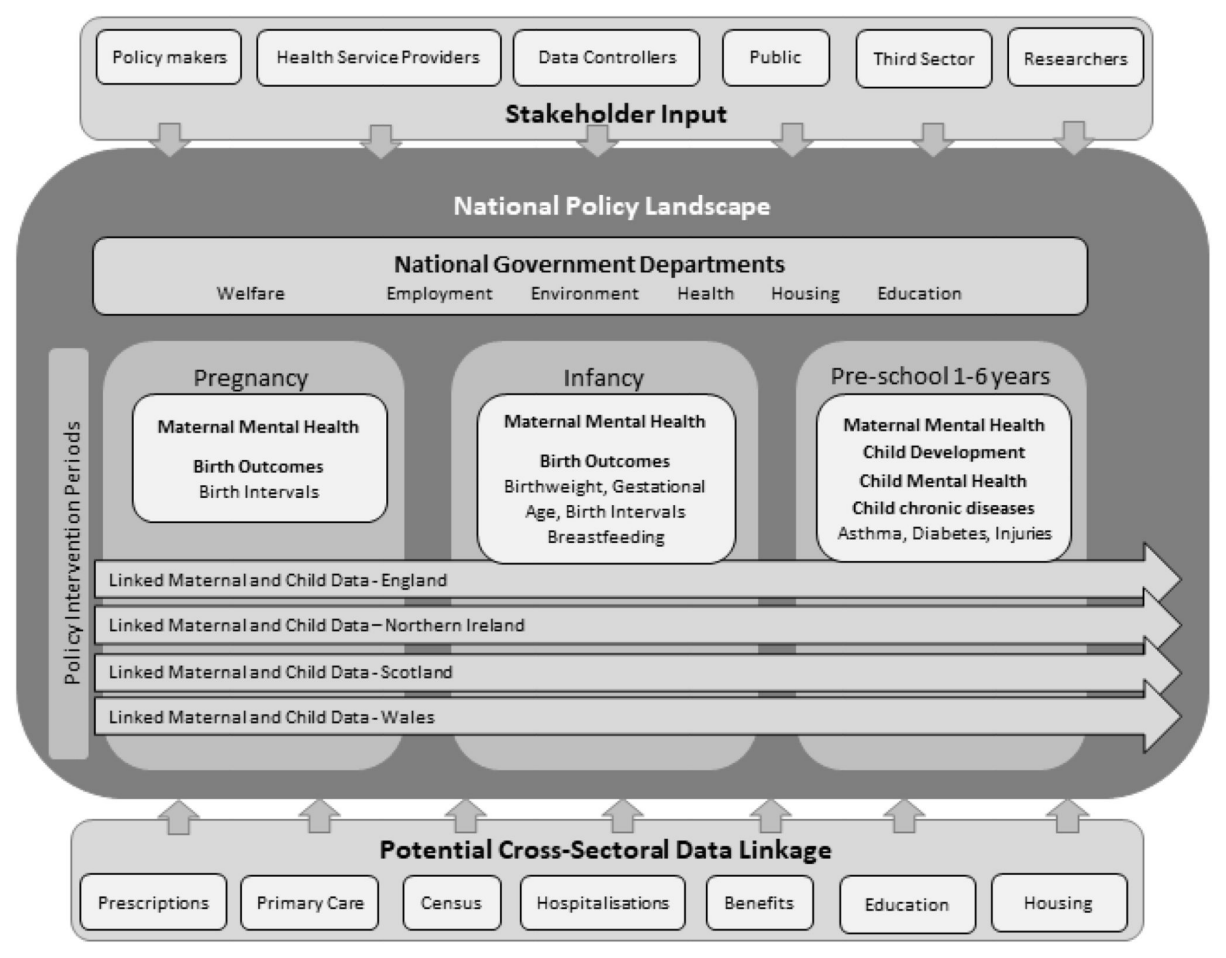
MatCHNet Framework - Policy intervention points, stakeholders, policy departments and longitudinal data sources and their integrations within the system

In 2019, the UK Prevention Research Partnership commissioned MatCHNet (Maternal and Child Health Network) to investigate how administrative data could be used to evaluate national policy impacts on child and maternal health. MatCHNet was required to identify a new programme of research. Initial consultation within the core network management group led to the development of the MatCHNet research framework and diagram. This identifies three key challenges that need to be addressed to evaluate early years interventions (see Box 1). The diagram (Figure 1) identifies policy intervention points, stakeholders, policy departments, longitudinal data sources and their integrations within the system necessary to address the three challenges.

Consultation on the research framework was conducted virtually due to the constraints of the Covid-19 pandemic. Feedback on the framework was provided via an online consultation (Summer 2021) as well as an online stakeholder roundtable discussion with policy makers, service providers, and third sector organisations. Comments and suggested amendments were provided on the three research challenges. This led to the framework being refined. Additional birth and pre-school outcomes were added as well as input from a range of different stakeholders.

Our framework identifies three key life periods where policy can intervene and have an impact on maternal and child health, leading to improvements in NCDs: pregnancy, infancy (0-1 year), and pre-school (1-6 years). Cutting across these three life periods are the three interlinked research challenges (see Box 1).

### Identifying policy priority areas in child and maternal health for future evaluation

First, there is a need to comprehensively map the numerous national interventions, across multiple policy domains and departments that can affect maternal and child health. Subsequently, national policy interventions and their contexts for evaluation should be prioritised. MatCHNet has sought multiple perspectives from policy makers, service providers and users, and researchers to identify policy priority areas. Options are constrained by the extent to which questions are evaluable, whether there are comparable data resources in multiple settings, similar measures within those data resources, and measurable variation in specific policies or contextual factors.

### Establishing what administrative longitudinal data can be linked and harmonised across the 4 UK countries

Second, data resources must be harmonised across the 4 UK nations to create consistent exposures and outcomes in the different settings. Longitudinal administrative health data for mothers and their children can be linked, which offers the potential for cross-country evaluation. In effect, whole country, longitudinal birth cohorts can be created in UK countries[23], with longitudinal records for children born up to 30 years ago (e.g., see Scottish Morbidity data from 1981). Such linkage provides information on maternal age at first birth, ethnicity, number of children, and through linkage to registration data, information on country of birth and parents’ relationship status. Further linkage to census data is now possible in Scotland, Wales, England, and for a proportion of the Northern Ireland population (28% sample in the Northern Ireland Longitudinal Study). This can provide important details about the household, migration status and employment. Mothers’ past education and children’s social care records can be linked to maternal health records for young mothers in some UK countries[24], and to the child at school entry. Linkage to survey data (e.g., the Scottish Health Survey; Health Survey for England; the UK Millennium Cohort Study) can add further information on social and environmental risk factors. Individual-level indicators of social and economic characteristics in administrative data are vital to evaluating interventions aimed at reducing child health inequalities in the early years[25].

### Determining suitable methods to evaluate national policies and make cross-country comparisons

Third, to undertake evaluations, it is necessary to identify the most appropriate methods to evaluate national policies within their specific contexts. For example, clearly defined and timed changes in policy could be evaluated using before vs after comparisons of health outcomes (interrupted time series analysis)[26–28]. A sudden change, such as introduction of universal free day care, might be suitable for regression discontinuity analysis. Both these examples can be used to evaluate national policies within a country, due to the occurrence of so-called ‘natural experiments’[24, 26]. Cross-country comparisons are valuable as these provide more confidence in the evaluation findings beyond natural experiments conducted solely within countries. Evaluations that compare policies and contexts between countries are at risk of bias if other important differences are also present, for example variations in eligibility thresholds for services. If these cannot be fully measured and adjusted for, then methods that combine natural experiments and cross-country comparisons (e.g., difference in difference studies) offer one solution.

All these methods not only come with different threats of bias but also different data requirements. For example, cross-country comparisons require comparable data over time and between countries, which may not always be possible. Evaluations should be undertaken according to best practice for natural experiment evaluations[21] and, therefore, consider triangulation of methods and analyses from multiple data resources. For example, augmenting administrative data with survey data or qualitative assessments from multiple settings.

## Summary

The proposed research framework outlines a blueprint for impactful evaluations of early years policy interventions. MatCHNet has several activities[29] aimed at tackling the three research challenges (see Box 2). Engagement with the policy sphere beyond health is essential to achieving policy impact and change that has meaningful impact upon children’s health and health inequalities. We hope this framework encourages new, cross-disciplinary, cross-national collaborations that will strengthen the evidence base in the field of child health research.

## Box 1 Child and maternal health research framework challenges

1. To identify priority national policies across the 4 UK nations that have most potential to affect maternal and child health.
2. To ascertain what longitudinal administrative data can be linked and harmonised across the 4 UK countries.
3. To determine suitable methods that can be used to evaluate national policies and make cross-country comparisons.

## Box 2 MatCHNet’s current and planned activities

- stakeholder roundtables with policy makers, service providers, and third sector organisations
- online research framework consultation
- policy mapping exercise
- online policy prioritisation poll
- consensus workshop with experts from academia
- policy briefing reports detailing UK variation in early years policies
- data mapping exercise
- methods masterclass workshops
